# Sustainable computational science: the ReScience initiative

**DOI:** 10.7717/peerj-cs.142

**Published:** 2017-12-18

**Authors:** Nicolas P. Rougier, Konrad Hinsen, Frédéric Alexandre, Thomas Arildsen, Lorena A. Barba, Fabien C.Y. Benureau, C. Titus Brown, Pierre de Buyl, Ozan Caglayan, Andrew P. Davison, Marc-André Delsuc, Georgios Detorakis, Alexandra K. Diem, Damien Drix, Pierre Enel, Benoît Girard, Olivia Guest, Matt G. Hall, Rafael N. Henriques, Xavier Hinaut, Kamil S. Jaron, Mehdi Khamassi, Almar Klein, Tiina Manninen, Pietro Marchesi, Daniel McGlinn, Christoph Metzner, Owen Petchey, Hans Ekkehard Plesser, Timothée Poisot, Karthik Ram, Yoav Ram, Etienne Roesch, Cyrille Rossant, Vahid Rostami, Aaron Shifman, Jemma Stachelek, Marcel Stimberg, Frank Stollmeier, Federico Vaggi, Guillaume Viejo, Julien Vitay, Anya E. Vostinar, Roman Yurchak, Tiziano Zito

**Affiliations:** 1 INRIA Bordeaux Sud-Ouest, Talence, France; 2 Centre de Biophysique Moléculaire UPR4301, CNRS, Orléans, France; 3 Department of Electronic Systems, Technical Faculty of IT and Design, Aalborg University, Aalborg, Denmark; 4 Department of Mechanical and Aerospace Engineering, The George Washington University, Washington, D.C., USA; 5 Department of Population Health and Reproduction, University of California Davis, Davis, CA, USA; 6 Instituut voor Theoretische Fysica, KU Leuven, Leuven, Belgium; 7 Laboratoire d’Informatique (LIUM), Le Mans University, Le Mans, France; 8 UNIC FRE 3693, CNRS, Gif-sur-Yvette, France; 9 Institut de Génétique et de Biologie Moléculaire et Cellulaire, Illkirch, France; 10 Department of Cognitive Sciences, University of California Irvine, Irvine, CA, USA; 11 Computational Engineering and Design, University of Southampton, Southampton, United Kingdom; 12 Humboldt Universität zu Berlin, Berlin, Germany; 13 Department of Neuroscience, Mount Sinai School of Medicine, New York, NY, USA; 14 Institute of Intelligent Systems and Robotics, Sorbonne Universités - UPMC Univ Paris 06 - CNRS, Paris, France; 15 Experimental Psychology, University College London, London, Greater London, United Kingdom; 16 UCL Great Ormond St Institute of Child Health, London, United Kingdom; 17 Champalimaud Centre for the Unknown, Champalimaud Neuroscience Program, Lisbon, Portugal; 18 Department of Ecology and Evolution, University of Lausanne, Lausanne, Switzerland; 19 Independent scholar, Enschede, The Netherlands; 20 BioMediTech Institute and Faculty of Biomedical Sciences and Engineering, Tampere University of Technology, Tampere, Finland; 21 Swammerdam Institute for Life Sciences, University of Amsterdam, Amsterdam, The Netherlands; 22 Department of Biology, College of Charleston, Charleston, SC, USA; 23 Centre for Computer Science and Informatics Research, University of Hertfordshire, Hatfield, United Kingdom; 24 Department of Evolutionary Biology and Environmental Studies, University of Zurich, Zurich, Switzerland; 25 Faculty of Science and Technology, Norwegian University of Life Sciences, Aas, Norway; 26 Département de Sciences Biologiques, Université de Montréal, Montréal, QC, Canada; 27 Berkeley Institute for Data Science, University of California, Berkeley, CA, USA; 28 Department of Biology, Stanford University, Stanford, CA, USA; 29 Centre for Integrative Neuroscience, University of Reading, Reading, United Kingdom; 30 Institute of Neurology, University College London, London, United Kingdom; 31 Institute of Neuroscience & Medicine, Juelich Forschungszentrum, Jülich, Germany; 32 Department of Biology, University of Ottawa, Ottawa, Ontario, Canada; 33 Department of Fisheries and Wildlife, Michigan State University, East Lansing, MI, USA; 34 Sorbonne Universités/UPMC Univ Paris 06/INSERM/CNRS/Institut de la Vision, Paris, France; 35 Max Planck Institute for Dynamics and Self-Organization, Göttingen, Lower Saxony, Germany; 36 Amazon, Seattle, WA, USA; 37 Department of Computer Science, Chemnitz University of Technology, Chemnitz, Saxony, Germany; 38 Department of Computer Science, Grinnell College, Grinnell, IA, USA; 39 Symerio, Palaiseau, France; 40 Neural Information Processing Group, Eberhard Karls Universität Tübingen, Tübingen, Germany

**Keywords:** Computational science, Open science, Publication, Reproducible, Replicable, Sustainable, GitHub, Open peer-review

## Abstract

Computer science offers a large set of tools for prototyping, writing, running, testing, validating, sharing and reproducing results; however, computational science lags behind. In the best case, authors may provide their source code as a compressed archive and they may feel confident their research is reproducible. But this is not exactly true. James Buckheit and David Donoho proposed more than two decades ago that an article about computational results is advertising, not scholarship. The actual scholarship is the full software environment, code, and data that produced the result. This implies new workflows, in particular in peer-reviews. Existing journals have been slow to adapt: source codes are rarely requested and are hardly ever actually executed to check that they produce the results advertised in the article. ReScience is a peer-reviewed journal that targets computational research and encourages the explicit replication of already published research, promoting new and open-source implementations in order to ensure that the original research can be replicated from its description. To achieve this goal, the whole publishing chain is radically different from other traditional scientific journals. ReScience resides on GitHub where each new implementation of a computational study is made available together with comments, explanations, and software tests.

## Introduction

There is a replication crisis in Science (Baker, 2016; Munafò et al., 2017). This crisis has been highlighted in fields as diverse as medicine (Ioannidis, 2005), psychology (Open Science Collaboration, 2015), the political sciences (Janz, 2015), and recently in the biomedical sciences (Iqbal et al., 2016). The reasons behind such non-replicability are as diverse as the domains in which it occurs. In medicine, factors such as *study power and bias, the number of other studies on the same question, and importantly, the ratio of true to no relationships among the all relationships probed* have been highlighted as important causes (Ioannidis, 2005). In psychology, non-replicability has been blamed on spurious *p*-values (p-hacking), while in the biomedical sciences (Iqbal et al., 2016), a *lack of access to full datasets and detailed protocols for both clinical and non-clinical biomedical investigation* is seen as a critical factor. The same remarks were recently issued for chemistry (Coudert, 2017). Surprisingly, the computational sciences (in the broad sense) and computer sciences (in the strict sense) are no exception (Donoho et al., 2009; Manninen, Havela & Linne, 2017) despite the fact they rely on code and data rather than on experimental observations, which should make them immune to the aforementioned problems.

When Colberg and colleagues (2016) decided to measure the extent of the problem precisely, they investigated the availability of code and data as well as *the extent to which this code would actually build with reasonable effort*. The results were dramatic: of the 515 (out of 613) potentially reproducible papers targeted by the study, the authors managed to ultimately run only 102 (less than 20%). These low numbers only reflect the authors’ success at running the code. They did not check for correctness of the code (i.e., does the code actually implement what is advertised in the paper), nor the reproducibility of the results (does each run lead to the same results as in the paper). One example of this problem can be found in Topalidou et al. (2015), in which the authors tried to replicate results obtained from a computational neuroscience model. Source code was not available, neither as supplementary material to the paper nor in a public repository. When the replicators obtained the source code after contacting the corresponding author, they found that it could not be compiled and would be difficult to reuse for other purposes.

Confronted with this problem, a small but growing number of journals and publishers have reacted by adopting explicit policies for data and software. Examples can be seen in the PLOS instructions on Materials and Software Sharing and on Data Availability, and in the recent announcement by eLife on forking (creating a linked copy of) software used in eLife papers to GitHub. Such policies help to ensure access to code and data in a well-defined format (Perkel, 2016) but this will not guarantee reproducibility nor correctness. At the educational and methodological levels, things have started to change with a growing literature on best practices for making computations reproducible (Sandve et al., 2013; Crook, Davison & Plesser, 2013; Wilson et al., 2014; Halchenko & Hanke, 2015; Janz, 2015; Hinsen, 2015). Related initiatives such as Software and Data Carpentry (Wilson, 2016) are of note since their goal is *to make scientists more productive, and their work more reliable, by teaching them basic computing skills*. Such best practices could be applied to already published research codebases as well, provided the original authors are willing to take on the challenge of re-implementing their software for the sake of better science. Unfortunately, this is unlikely since the incentives for doing such time-consuming work are low or nonexistent. Furthermore, if the original authors made mistakes in their original implementation, it seems likely that they will reproduce their mistakes in any re-implementation.

## Replication and Reproduction

While recognition of the replication crisis as a problem for scientific research has increased over time, unfortunately no common terminology has emerged so far. One reason for the diverse use of terms is that each field of research has its own specific technical and social obstacles on the road to publishing results and findings that can be verified by other scientists. Here we briefly summarize the obstacles that arise from the use of computers and software in scientific research, and introduce the terminology we will use in the rest of this article. We note, however, that there is some disagreement about this particular choice of terminology even among the authors of this article.

*Reproducing* the result of a computation means running the same software on the same input data and obtaining the same results. The goal of a reproduction attempt is to verify that the computational protocol leading to the results has been recorded correctly. Performing computations reproducibly can be seen as a form of provenance tracking, the software being a detailed record of all data processing steps.

In theory, computation is a deterministic process and exact reproduction should therefore be trivial. In reality, it is very difficult to achieve because of the complexity of today’s software stacks and the tediousness of recording all interactions between a scientist and a computer (although a number of recent tools have attempted to automate such recording, e.g., Guo & Engler, 2011; Davison, 2012; Murta et al., 2015). Mesnard and Barba explain (Mesnard & Barba, 2017) how difficult it can be to reproduce a two-year-old computation even though all possible precautions were taken at the time to ensure reproducibility. The most frequent obstacles are the loss of parts of the software or input data, lack of a computing environment that is sufficiently similar to the one used initially, and insufficient instructions for making the software work. An obstacle specific to numerical computations is the use of floating-point arithmetic, whose rules are subject to slightly different interpretations by different compilers and runtime support systems. A large variety of research practices and support tools have been developed recently to facilitate reproducible computations. For a collection of recipes that have proven useful, see Kitzes, Turek & Deniz (2017).

Publishing a reproducible computational result implies publishing all the software and all the input data, or references to previously published software and data, along with the traditional article describing the work. An obvious added value is the availability of the software and data, which helps readers to gain a better understanding of the work, and can be re-used in other research projects. In addition, reproducibly published results are more trustworthy, because many common mistakes in working with computers can be excluded: mistyping parameter values or input file names, updating the software but forgetting to mention the changes in the description of the method, planning to use one version of some software but actually using a different one, etc.

Strictly speaking, reproducibility is defined in the context of identical computational environments. However, useful scientific software is expected to be robust with respect to certain changes in this environment. A computer program that produces different results when compiled using different compilers, or run on two different computers, would be considered suspect by most practitioners, even if it were demonstrably correct in one specific environment. Ultimately it is not the software that is of interest for science, but the models and methods that it implements. The software is merely a vehicle to perform computations based on these models and methods. If results depend on hard-to-control implementation details of the software, their relation to the underlying models and methods becomes unclear and unreliable.

*Replicating* a published result means writing and then running new software based on the description of a computational model or method provided in the original publication, and obtaining results that are similar enough to be considered equivalent. What exactly “similar enough” means strongly depends on the kind of computation being performed, and can only be judged by an expert in the field. The main obstacle to replicability is an incomplete or imprecise description of the models and methods.

Replicability is a much stronger quality indicator than reproducibility. In fact, reproducibility merely guarantees that all the ingredients of a computation are well documented. It does not imply that any of them are correct and/or appropriate for implementing the models and methods that were meant to be applied, nor that the descriptions of these models and methods are correct and clear. A successful replication shows that two teams have produced independent implementations that generate equivalent results, which makes serious mistakes in either implementation unlikely. Moreover, it shows that the second team was able to understand the description provided by the first team.

Replication can be attempted for both reproducible and non-reproducible results. However, when an attempt to replicate non-reproducible work fails, yielding results too different to be considered equivalent, it can be very difficult to identify the cause of the disagreement. Reproducibility guarantees the existence of a precise and complete description of the models and methods being applied in the original work, in the form of software source code, which can be analyzed during the investigation of any discrepancies. The holy grail of computational science is therefore a reproducible replication of reproducible original work.

## The ReScience Initiative

Performing a replication is a daunting task that is traditionally not well rewarded. Nevertheless, some people are willing to replicate computational research. The motivations for doing so are very diverse (see Box 1). Students may want to familiarize themselves with a specific scientific domain, and acquire relevant practical experience by replicating important published work. Senior researchers may critically need a specific piece of code for a research project and therefore re-implement a published computational method. If these people write a brand new open source implementation of already published research, it is likely that this new implementation will be of interest for other people as well, including the original authors. The question is where to publish such a replication. To the best of our knowledge, no major journal accepts replications in computational science for publication. This was the main motivation for the creation of the ReScience journal (https://rescience.github.io) by Konrad Hinsen and Nicolas P. Rougier in September 2015.

BOX 1 Authors having published in Rescience explain their motivation.

**(Stachelek, 2016)** I was motivated to replicate the results of the original paper because I feel that working through code supplements to blog posts has really helped me learn the process of scientific analysis. I could have published my replication as a blog post but I wanted the exposure and permanency that goes along with journal articles. This was my first experience with formal replication. I think the review was useful because it forced me to consider how the replication would be used by people other than myself. I have not yet experienced any new interactions following publication. However, I did notify the author of the original implementation about the replication’s publication. I think this may lead to future correspondence. The original author suggested that he would consider submitting his own replications to ReScience in the future.

**(Topalidou & Rougier, 2015)** Our initial motivation and the main reason for replicating the model is that we needed it in order to collaborate with our neurobiologist colleagues. When we arrived in our new lab, the model had just been published (2013) but the original author had left the lab a few months before our arrival. There was no public repository nor version control, and the paper describing the model was incomplete and partly inaccurate. We managed to get our hands on the original sources (6,000 lines of Delphi) only to realize we could not compile them. It took us three months to replicate it using 250 lines of Python. But at this time, there was no place to publish this kind of replication to share the new code with colleagues. Since then, we have refined the model and made new predictions that have been confirmed. Our initial replication effort really gave the model a second life.

**(Viejo, Girard & Khamassi, 2016)** Replicating previous work is a relatively routine task every time we want to build a new model: either because we want to build on this previous work, or because we want to compare our new model to it. We also give replication tasks to M.Sc. students every year, as projects. In all these cases, we are confronted with incomplete or inaccurate model descriptions, as well as with the impossibility to obtain the original results. Contacting the original authors sometimes solves the problem, but not so often (because of the *dog ate my hard drive* syndrome). We thus accumulate knowledge, internal to the lab, about which model works and which doesn’t, and how a given model has to be parameterized to really work. Without any place to publish it, this knowledge is wasted. Publishing it in ReScience, opening the discussion publicly, will be a progress for all of us.

ReScience is an openly-peer-reviewed journal that targets computational research and encourages the explicit replication of already published research. In order to provide the largest possible benefit to the scientific community, replications are required to be reproducible and open-source. In two years of existence, 17 articles have been published and 4 are currently under review (#20, #39, #41, #43). The editorial board covers a wide range of computational sciences (see http://rescience.github.io/board/) and more than 70 volunteers have registered to be reviewers. The scientific domains of published work are computational neuroscience, neuroimaging, computational ecology and computer graphics, with a majority in computational neuroscience. The most popular programming languages are Python and R. The review process takes about 100 days on average and involves about 50 comments. There is a strong bias towards successful replication (100%); experience has taught us that researchers are reluctant to publish failed replications, even when they can prove that the original work is wrong. For young researchers, there is a social/professional risk in publishing articles that show results from a senior researcher to be wrong. Until we implement a certified anonymized submission process, this strong bias will most likely remain.

One of the specificities of the ReScience journal is a publishing chain that is radically different from any other traditional scientific journal, since ReScience lives on GitHub, a platform originally designed for collaborative software development. A ReScience submission is treated very similarly to a contribution to an Open Source software project. One of the consequences is that the whole process, from submission via reviewing to publication, is open for anyone to see and even comment on.

Each submission is considered by a member of the editorial board, who may decide to reject the submission if it does not respect the formal publication criteria of ReScience. A submission must contain

• a precise reference to the work being replicated,
• an explanation of why the authors think they have replicated the paper (same figures, same graphics, same behavior, etc.) or why they have failed,
• a description of any difficulties encountered during the replication,
• open-source code that produces the replication results,
• an explanation of this code for human readers.

A complete submission therefore consists of both computer code and an accompanying article, which are sent to ReScience in the form of a pull request (the process used on GitHub to submit a proposed modification to a software project). Partial replications that cover only some of the results in the original work are acceptable, but must be justified.

If the submission respects these criteria, the editor assigns it to two reviewers for further evaluation and tests. The reviewers evaluate the code and the accompanying material in continuous interaction with the authors through the discussion section until both reviewers consider the work acceptable for publication. The goal of the review is thus to help the authors meet the ReScience quality standards through discussion. Since ReScience targets replication of already published work, the criteria of importance or novelty applied by most traditional journals are irrelevant.

For a successful submission (i.e., partial or full replication) to be accepted, both reviewers must consider it reproducible and a valid replication of the original work. As we explained earlier, this means that the reviewers

• are able to run the proposed implementation on their computers,
• obtain the same results as indicated in the accompanying paper,
• consider these results sufficiently close to the ones reported in the original paper being replicated.

For a failure to replicate submission to be accepted, we require extra steps to be taken. In addition to scrutiny of the submission by reviewers and editors, we will try to contact the authors of the original research, and issue a challenge to the community to spot and report errors in the new implementation. If no errors are found, the submission will be accepted and the original research will be declared non-replicable.

Since independent implementation is a major feature of replication work, ReScience does not allow authors to submit replications of their own research, nor the research of close collaborators. Moreover, replication work should be based exclusively on the originally published paper, although exceptions are admitted if properly documented in the replication article. Mistakes in the implementation of computational models and methods are often due to biases that authors invariably have, consciously or not. Such biases will inevitably carry over to a replication. Perhaps even more importantly, cross-fertilization is generally useful in research, and trying to replicate the work of one’s peers might pave the way for a future collaboration, or may give rise to new ideas as a result of the replication effort.

## Lessons Learned

Although ReScience is still a young project, the submissions handled so far already provide valuable experience concerning the reproducibility and replicability of computational work in scientific research.

### Short-term and long-term reproducibility

While some of the reasons for non-reproducibility are specific to each scientific domain, our experience has shown that there are also some common issues that can be identified. Missing code and/or data, undocumented dependencies, and inaccurate or imprecise description appear to be characteristic of much non-reproducible work. Moreover, these problems are not always easy to detect even for attentive reviewers, as we discovered when some articles published in ReScience turned out to be difficult to reproduce for someone else for exactly the reasons listed above. ReScience reviewers are scientists working in the same domain as the submitting authors, because familiarity with the field is a condition for judging if a replication is successful. But this also means that our reviewers share a significant common background with the authors, and that background often includes the software packages and programming languages adopted by their community. In particular, if both authors and reviewers have essential libraries of their community installed on their computers, they may not notice that these libraries are actually dependencies of the submitted code. While solutions to this problem evidently exist (ReScience could, for example, request that authors make their software work on a standard computational environment supplied in the form of a virtual machine), they represent an additional effort to authors and therefore discourage them from submitting replication work to ReScience. Moreover, the evaluation of *de-facto* reproducibility (“works on my machine”) by reviewers is useful as well, because it tests the robustness of the code under small variations in the computational environments that are inevitable in real life. Our goal is to develop a set of recommendations for authors that represent a workable compromise between reproducibility, robustness, and implementation effort. These recommendations will evolve over time, and we hope that with improving technology we will ultimately reach full reproducibility over a few decades.

Another issue with reproducibility is that with today’s computing technology, long-term reproducibility can only be achieved by imposing drastic constraints on languages and libraries that are not compatible with the requirements of research computing. This problem is nicely illustrated by Mesnard & Barba (2017) whose authors report trying to reproduce their own work performed two years earlier. Even though Barba’s group is committed to reproducible research practices, they did not escape the many problems one can face when trying to re-run a piece of code. As a consequence, code that is written for ReScience today will likely cease to be functional at some point in the future. The long-term value of a ReScience publication lies not just in the actual code but also in the accompanying article. The combination of the original article and the replication article provide a complete and consistent description of the original work, as evidenced by the fact that replication was possible. Even 5, 10, or 20 years later, a competent scientist should be able to replicate the work again thanks to these two articles. Of course, the new code can also help, but the true long-term value of a replication is the accompanying article.

### Open reviewing

The well-known weaknesses of the traditional anonymous peer-reviewing system used by most scientific journals have motivated many experiments with alternative reviewing processes. The variant adopted by ReScience is similar to the ones used by F1000Research or PeerJ, but is even more radically open: anyone can look at ReScience submissions and at the complete reviewing process, starting from the assignment of an editor and the invitation of reviewers. Moreover, anyone with a GitHub account can intervene by commenting. Such interventions could even be anonymous because a GitHub account is not required to advertise a real name or any other identifying element. ReScience does currently require all authors, editors, and reviewers to provide real names (which however are not verified in any way), but there are valid reasons to allow anonymity for authors and reviewers, in particular to allow junior scientists to criticize the work of senior colleagues without fear of retribution, and we envisage exploring such options in the future.

Our experience with this open reviewing system is very positive so far. The exchanges between reviewers and authors are constructive and courteous, without exception. They are more similar in style to a coffee-table discussion than to the judgement/defence style that dominates traditional anonymous reviewing. Once reviewers have been invited and have accepted the task, the editors’ main role is to ensure that the review moves forward, by gently reminding everyone to reply within reasonable delays. In addition, the editors occasionally answer questions by authors and reviewers about the ReScience publishing process.

The possibility to involve participants beyond the traditional group of authors, editors, and reviewers is particularly interesting in the case of ReScience, because it can be helpful to solicit input from the authors of the original study that is being replicated. For example, in one recent case (#28), a reviewer suggested asking the author of the original work for permission to re-use an image. The author intervened in the review and granted permission.

### Publishing on the GitHub platform

GitHub is a commercial platform for collaborative software development based on the popular version control system git. It offers unlimited free use to public projects, defined as projects whose contents are accessible to everyone. All ReScience activities are organized around a few such Open Source projects hosted by GitHub. This is an unusual choice for a scientific journal, the only other journal hosted on GitHub being The Journal of Open Source Software (Smith et al., 2017). In this section, we discuss the advantages and problems resulting from this choice, considering both technical and social issues.

There are clear differences between platforms for software development, such as GitHub, and platforms for scientific publishing, such as HighWire. The latter tend to be expensive commercial products developed for the needs of large commercial publishers, although the market is beginning to diversify with products such as Episciences. More importantly, to the best of our knowledge, no existing scientific publishing platform supports the submission and review of code, which is an essential part of every ReScience article. For this reason, the only option for ReScience was to adopt a software development platform and develop a set of procedures that make it usable for scientific publishing.

Our experience shows that the GitHub platform provides excellent support for the reviewing process, which is not surprising given that the review of a scientific article containing code is not fundamentally different from the review of code with accompanying documentation. One potential issue for other journals envisaging adoption of this platform is the necessity that submitting authors have a basic knowledge of the version control system Git and of the techniques of collaborative software development. Given the code-centric nature of ReScience, this has not been a major problem for us, and the minor issues have been resolved by our editors providing technical assistance to authors. It is of course possible that potential authors are completely discouraged from submitting to ReScience by their lack of the required technical competence, but so far nobody has provided feedback suggesting that this is a problem.

The main inconvenience of the GitHub platform is its almost complete lack of support for the publishing steps, once a submission has successfully passed the reviewing process. At this point, the submission consists of an article text in Markdown format plus a set of code and data files in a git repository. The desired archival form is an article in PDF format plus a permanent archive of the submitted code and data, with a Digital Object Identifier (DOI) providing a permanent reference. The Zenodo platform allows straightforward archiving of snapshots of a repository hosted on GitHub, and issues a DOI for the archive. This leaves the task of producing a PDF version of the article, which is currently handled by the managing editor of the submission, in order to ease the technical burden on our authors.

A minor inconvenience of the GitHub platform is its implementation of code reviews. It is designed for reviewing contributions to a collaborative project. The contributor submits new code and modifications to existing code in the form of a “pull request”, which other project members can then comment on. In the course of the exchanges, the contributor can update the code and request further comments. Once everybody is satisfied, the contribution is “merged” into the main project. In the case of ReScience, the collaborative project is the whole journal, and each article submission is a contribution proposed as a pull request. This is, however, not a very intuitive representation of how a journal works. It would be more natural to have a separate repository for each article, an arrangement that would also facilitate the final publishing steps. However, GitHub does not allow code review on a new repository, only on contributions to an already existing one.

Relying on a free-use offer on a commercial platform poses some additional problems for scientific publishing. GitHub can change its conditions at any time, and could in principle delete or modify ReScience contents at any time without prior notice. Moreover, in the case of technical problems rendering ReScience contents temporarily or permanently inaccessible, the ReScience community has no legal claims for compensation because there is no contract that would imply any obligations for GitHub. It would clearly be imprudent to count on GitHub for long-term preservation of ReScience content, which is why we deposit accepted articles on Zenodo, a platform designed for archiving scientific information and funded by research organizations as an element of public research infrastructure.

The use of free services provided by GitHub and Zenodo was clearly important to get ReScience started. The incentives for the publication of replication work being low, and its importance being recognized only slowly in the scientific community, funding ReScience through either author page charges or grants would have created further obstacles to its success. A less obvious advantage of not having to organize funding is that ReScience can exist without being backed by any legal entity that would manage its budget. This makes it possible to maintain a community spirit focused on shared scientific objectives, with nobody in a position to influence ReScience by explicit or implicit threats of reducing future funding.

## Outlook

Based on our experience with the ReScience initiative, we can engage in informed speculation about possible future evolutions in scientific publishing, in particular concerning replication work. We will not discuss minor technical advances such as a better toolchain for producing PDF articles, but concentrate on long-term improvements in the technology of electronic publishing and, most of all, in the attitude of the scientific community towards the publication, preservation, and verification of computer-aided research.

A fundamental technical issue is the difficulty of archiving or accurately describing the software environments in which computational scientists perform their work. A publication should be accompanied by both a human-readable description of this environment and an executable binary form. The human-readable description allows an inspection of the versions of all software packages that were used, for example to check for the impact of bugs that become known only after a study was published. The executable version enables other scientists to re-run the analyses and inspect intermediate results. Ideally, the human-readable description would permit rebuilding the executable version, in the same way that software source code permits rebuilding executable binaries. This approach is pursued for example by the package manager Guix (Courtés & Wurmus, 2015). A more limited but still useful implementation of the same idea exists in the form of the conda package manager (Anaconda Inc., 2017), which uses a so-called environment file to describe and reconstruct environments. The main limitation compared to Guix is that the packages that make up a conda environment are themselves not reproducible. For example, a conda environment file does not state which compiler versions were used to build a package.

Containerization, as implemented e.g., by Docker (Docker Inc., 2017) is currently much discussed, but provides only the executable version without a human-readable description. Moreover, the long-term stability of the container file format remains to be evaluated. History has shown that long-term stability in computing technology is achieved only by technology for which it is a design priority, as in the case of the Java Virtual Machine (Lindholm & Yellin, 1999). Docker, on the contrary, is promoted as a deployment technology with no visible ambition towards archiving of computational environments.

Today’s electronic publishing platforms for scientific research still show their origins in paper-based publishing. Except for the replacement of printed paper by a printable PDF file, not much has changed. Although it is increasingly realized that software and data should be integral parts of most scientific publications today, they are at best relegated to the status of “supplementary material”, and systematically excluded from the peer review process. In fact, to the best of our knowledge, ReScience is the only scientific journal that aims to verify the correctness of scientific software. As our experience has shown, it is far easier to graft publication onto a software development platform than to integrate software reviewing into a publishing platform. Furthermore, tools that will allow for the automated validation of computational models and the automated verification of correctness are being actively developed in the community (see, for example, SciUnit or OSB-model-validation). An integration of such frameworks, which would greatly enhance the verification and validation process, seems feasible for the existing software development platforms.

**Figure 1:**
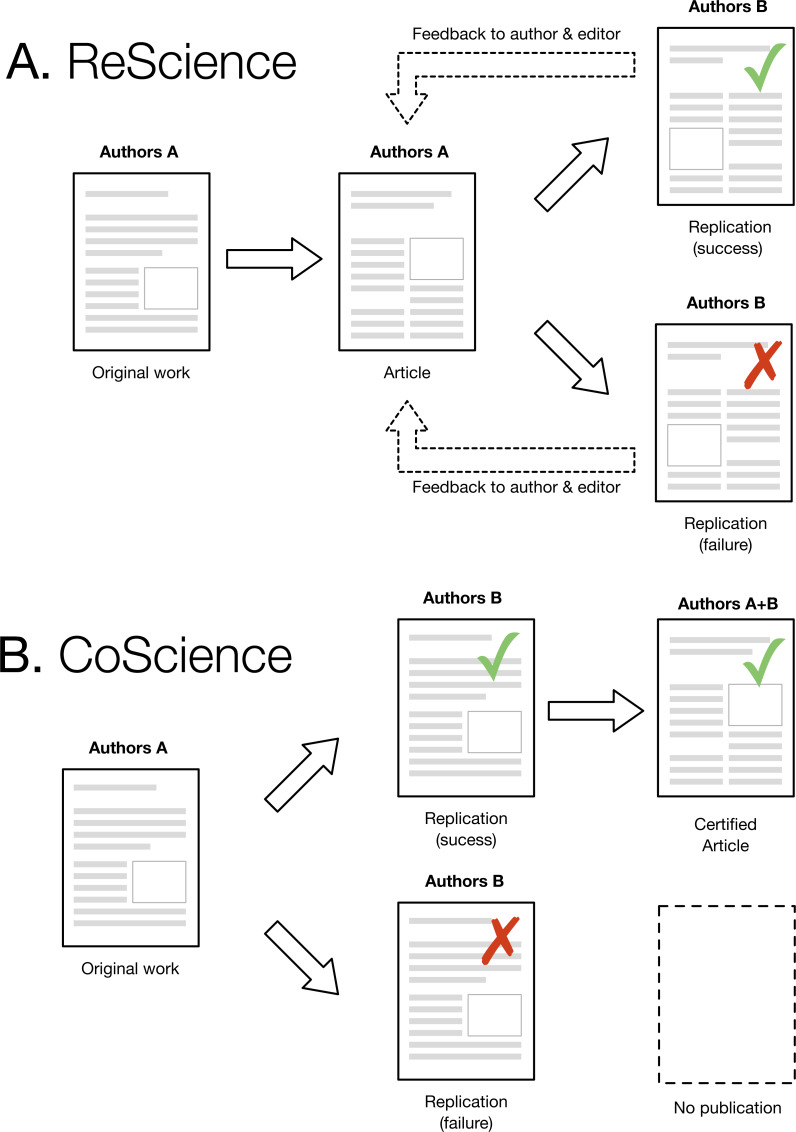
(A) The ReScience publication chain starts from an original research article by authors A, published in a journal, in conference proceedings, or as a preprint. This article constitutes the base material for authors B, who attempt to replicate the work based on its description. Success or failure to replicate is not a criterion for acceptance or rejection, even though failure to replicate requires more precaution to ensure this is not a misunderstanding or a bug in the new code. After review, the replication is published, and feedback is given to original authors (and editors) to inform them the work has been replicated (or not). (B) The CoScience proposal would require the replication to happen *before* the actual publication. In case of failure, nothing will be published. In case of success, the publication will be endorsed by authors A and authors B with identified roles and will be certified as reproducible because it has been replicated by an independent group.

A logical next step is to fully embrace the technology designed for software development, which far better takes into account the specificity of electronic information processing than today’s scientific publishing systems. In addition to the proper handling of code, such an approach offers further advantages. Perhaps the most important one is a shift of focus from the paper as a mostly isolated and finished piece of work to scientific progress as a collection of incremental and highly interdependent steps. The Software Heritage project, whose aim is to create a permanent public archive of all publicly available software source code, adopts exactly this point of view for the preservation of software. As our experience with ReScience has shown, integrating the narrative of a scientific article into a framework designed for software development is not difficult at all. Publishing and archiving scientific research in Software Heritage would offer several advantages. The intrinsic identifiers that provide access to the contents of the archive permit unambiguous and permanent references to ongoing projects as well as to snapshots at a specific time, and to whole projects as well as to the individual files that are part of them. Such references hold the promise for better reuse of scientific information, for better reproducibility of computations, and for fairer attribution of credit to scientists who contribute to research infrastructure.

One immediate and legitimate question is to wonder to what extent a replication could be performed rior to the publication of the original article. This would strongly reinforce a claim because a successful and independent replication would be available right from the start. As illustrated in Fig. 1, this would require group A to contact group B and send them a draft of their original work (the one that would be normally submitted to a journal) such that group B could perform a replication and confirm or refute the results. In case of confirmation, a certified article could be later published with both groups as authors (each group being identified according to their respective roles). However, if the replication fails and the original work cannot be fixed, this would prevent publication. This model would improve the quality of computational research and also considerably slow down the rapid pace of publication we are observing today. Unfortunately, such a scenario seems highly improbable today. The pressure to publish is so strong and the incentive for doing replication so low that it would most probably prevent such collaborative work. However, we hope that the current replication crisis will lead to a change in attitude, with an emphasis on the quality rather than the quantity of scientific ouput, with CoScience becoming the gold-standard approach to quality assurance.

## Additional Information and Declarations

### Data Availability

The following information was supplied regarding data availability: ReScience journal: https://zenodo.org/communities/rescience/.
